# The impact of physical exercise on the social adaptability of college students: the chain mediating role of peer relationships and psychological resilience

**DOI:** 10.3389/fpsyg.2026.1885215

**Published:** 2026-07-23

**Authors:** Shuochen Zhang, Hongbo Zhao

**Affiliations:** School of Physical Education, Liaoning Normal University, Dalian, China

**Keywords:** peer relationships, physical exercise, postgraduate students, psychological resilience, social adaptation ability

## Abstract

**Objective:**

This study aims to investigate the impact of physical exercise on college students’ social adaptation abilities and to analyze the chained mediating effects of peer relationships and psychological resilience, thereby providing a theoretical foundation and practical guidance for college students to better enhance their social adaptation abilities.

**Methods:**

A convenience sampling method was used to conduct a survey of college students at selected universities in Liaoning, Jilin, and Heilongjiang provinces in China. Data were collected using the Physical Activity Level Scale, the Social Adaptation Ability Diagnostic Scale, the Peer Relationship Scale, and the Psychological Resilience Scale. A total of 861 questionnaires were distributed, with 861 returned; of these, 802 were valid, resulting in a response rate of 93.15%. Subsequently, SPSS was used to analyze the relationships among variables, and a structural equation model was constructed using AMOS 29.0.

**Results:**

(1) Physical exercise is significantly positively correlated with college students’ social adaptation ability; (2) Physical exercise positively predicts peer relationships, psychological resilience, and college students’ social adaptation ability; peer relationships positively predict psychological resilience and college students’ social adaptation ability; psychological resilience positively predicts college students’ social adaptation ability; (3) Peer relationships and psychological resilience act as independent mediators between physical exercise and college students’ social adaptation; (4) Peer relationships and psychological resilience act as chain mediators between physical exercise and college students’ social adaptation.

**Implications:**

Physical exercise not only has a direct positive impact on college students’ social adaptation abilities, but also indirectly enhances these abilities through the chain mediation of peer relationships and psychological resilience.

## Introduction

Currently, mental health issues such as depression and anxiety among Chinese college students have become increasingly prominent (Yu, 2023). In January 2025, the Central Committee of the Communist Party of China and the State Council issued the “Outline of the Plan for Building a Strong Nation in Education (2024–2035),” which calls for enhancing students’ practical skills, ability to solve complex problems, and social adaptability, thereby promoting their healthy growth and all-round development (Central Committee of the Communist Party of China, State Council, 2025). Social adaptation, as a key element connecting the individual to the environment, has a decisive impact on both the current academic performance and future career development of college students (Shan, 2025). However, with the deepening development of the digital age, an increasing number of students prefer to engage in online socializing from home, neglecting participation in offline outdoor activities. This shift in lifestyle and social interaction has led contemporary college students to encounter numerous challenges in their social adaptation process, specifically manifested in issues such as strained interpersonal relationships in dormitories and weak emotional regulation skills (Zhang and Zhang, 2007). This directly results in a weakening of students’ social adaptation abilities, making it difficult for them to adapt to complex social environments and successfully transition into their social roles after graduation.

Contemporary college students are currently in a critical transitional phase as they prepare to enter society, where they will soon face intense job competition and a fast-paced social environment; enhancing their social adaptability is now a matter of urgency. Physical exercise is widely recognized as a key component of personal health; it not only strengthens physical fitness and promotes cognitive, emotional, and social development, but also helps individuals form a correct sense of self, self-awareness, and self-discovery, thereby fostering personality development and facilitating socialization (Li, 2007). The Positive Emotion Expansion-Construction Theory posits that positive emotions can expand the scope of an individual’s cognitive and social behaviors (expansion effect) and build psychological and social capital (construction effect), thereby continuously enhancing an individual’s comprehensive ability to adapt to the environment, cope with stress, and integrate into society (Fredrickson, 2004). Physical exercise can evoke positive emotions such as joy, satisfaction, and pride, enabling students to participate in social activities with a positive attitude. These positive emotions strengthen peer relationships by fostering trust and mutual respect through interaction (Xing and Ge, 2025), and healthy peer relationships can significantly enhance students’ interpersonal skills, promote the development of their empathy, and thereby strengthen their social adaptability (Allen et al., 2005), fulfilling the expansion effect; at the same time, long-term, sustained positive emotions can offset the negative impact of negative emotions, helping to build enduring and stable psychological and social resources for the individual. This forms psychological protective capital, enhances an individual’s ability to cope with stress, and consequently improves psychological resilience (Gao and Tong, 2010). University students with greater psychological resilience tend to view problems more positively, reducing the likelihood of negative outcomes (Lasota and Mroz, 2021). This enables them to better regulate their emotions, utilize their social support systems more effectively, and adopt effective coping strategies to adapt to new environments (Luo et al., 2018), thereby improving their social adaptability (Fan et al., 2026), which fulfills the construction effect.

Based on this, this study adopts the Positive Emotion Expansion-Construction Theory as its analytical framework. Against the backdrop of enhancing college students’ social adaptation abilities, it aims to thoroughly investigate the impact of physical exercise on these abilities, as well as the chained mediating roles of peer relationships and psychological resilience between the two, thereby providing a new theoretical basis for improving college students’ social adaptation abilities.

### Theoretical framework and assumptions

#### The relationship between physical exercise and social adaptability among college students

Physical exercise, also known as physical activity, refers to planned, organized, and repetitive physical activities aimed at enhancing physical fitness and improving physical and mental health (Liu, 2006). Social adaptation refers to an individual’s ability to effectively cope with daily life and fulfill social responsibilities appropriate to their age group and sociocultural environment (Kapp, 2023). Existing research indicates that personal circumstances, physical activity participation, and family background have a direct impact on college students’ social adaptation, while campus sports culture, sports environment, and sports-related interactions have a positive influence on their social adaptation abilities (Li, 2009). Additionally, studies have demonstrated a significant positive correlation between physical exercise and college students’ social adaptation abilities, with physical exercise serving as a significant predictor of these abilities (Yang, 2024). The greater the amount of physical exercise, the stronger the social adaptation ability. Regular physical activity not only significantly enhances an individual’s physical fitness but also effectively improves students’ psychological well-being; this dual-promoting effect enables participants to better cope with various pressures and challenges in life (Tian, 2024).

Therefore, we propose Hypothesis H1: Physical exercise has a positive effect on college students’ social adaptation abilities.

### The mediating role of peer relationships

Peer relationships refer to interpersonal bonds formed between individuals of similar age and developmental level, characterized by equality, voluntary interaction, and mutual influence. As horizontal interpersonal relationships distinct from kinship and authority-based relationships, they play a central role in an individual’s socialization, emotional support, and the formation of self-concept (Rubin et al., 2008). Physical exercise and peer relationships exhibit a significant positive correlation; active participation in physical activities can enhance college students’ peer relationships (Ma and Shi, 2022). Research indicates that physical exercise can maintain and improve the quality of friendships with others, leading to greater support and acceptance of individual behavior and enhancing self-esteem (Chen and Yu, 2015). Physical activities provide adolescents with numerous opportunities for equal interaction. Long-term, regular physical exercise can continuously optimize adolescents’ interpersonal communication styles, fostering peer companionship, trust, and group cohesion (Weiss, 1992). Adolescents participating in physical activities are immersed in a positive atmosphere during exercise; through sustained physical interaction and cooperation, the frequency and quality of their peer interactions are significantly enhanced, thereby promoting the formation of healthy peer relationships (Qin and Qin, 2023). At the same time, research indicates that the quality of students’ peer relationships is significantly positively correlated with their social adaptation abilities. Strong peer relationships provide students with a “safe haven,” enabling them to seek help, share experiences, and cope with stress—all of which are crucial for their emotional stability and social development (Meeus et al., 2005). Furthermore, as time spent interacting with peers at school accumulates, the impact of these relationships on individual development gradually deepens, ultimately becoming a key predictor of social adaptation levels (Zhou, 2024).

It follows that physical exercise may influence college students’ social adjustment through peer relationships; therefore, we propose Hypothesis H2: Peer relationships may mediate the relationship between physical exercise and social adjustment.

### The mediating role of psychological resilience

Psychological resilience refers to an individual’s psychological capacity and process for coping, regulating, and adapting effectively when faced with trauma, adversity, difficulties, and significant life stressors (Luthar and Cicchetti, 2000); it is also known as “resilience,” “psychological resilience,” or “psychological elasticity.” Research indicates that regular physical exercise can significantly and positively predict and enhance an individual’s level of psychological resilience (Szuhany et al., 2023). Physical exercise is positively correlated with psychological resilience. Students with low exercise intensity exhibit the lowest levels of psychological resilience, followed by those with high exercise intensity, while students with moderate exercise intensity demonstrate the highest levels. Students who frequently engage in physical exercise have higher levels of psychological resilience than those who do not (Cheng, 2021); that is, the higher the level of physical exercise among college students, the better their psychological resilience (Liu, 2023). At the same time, research also indicates that college students’ psychological resilience is significantly positively correlated with their social adaptation abilities (Wang, 2013). Students with superior resilience demonstrate strong initiative in balancing academic development with daily routines; their deep-seated independent will and sense of efficacy are significantly enhanced as a result. When facing adversity, they are able to effectively self-regulate and adopt proactive coping strategies while maintaining an optimistic and open-minded attitude, thereby reducing their perception of stress and promoting rapid improvement in social adaptation abilities (Yuan, 2019).

It follows that physical exercise may influence college students’ social adaptation through psychological resilience; therefore, we propose Hypothesis H3: Psychological resilience mediates the relationship between physical exercise and social adaptation.

### The chain mediation of peer relationships and psychological resilience

Research has found that peer relationships can significantly and positively predict psychological resilience (Ren, 2021), and there is a significant positive correlation between peer relationships and psychological resilience (Shan, 2017). Harmonious and positive peer relationships and peer support can lead to greater emotional stability, reduced stress, and increased self-confidence (Wu and Man, 2018). Good peer relationships are an important external protective factor for psychological resilience; the more peer support adolescents receive, the stronger their ability to adapt to adversity and the higher their level of psychological resilience (Hu and Gan, 2008). Conversely, poor peer relationships, such as peer rejection and social disadvantage, can significantly reduce psychological resilience (Sentse and Prinstein, 2017).

Based on this, this study proposes Hypothesis H4: Peer relationships and psychological resilience mediate the relationship between physical exercise and social adaptation in a chain-like manner.

**Figure 1:**
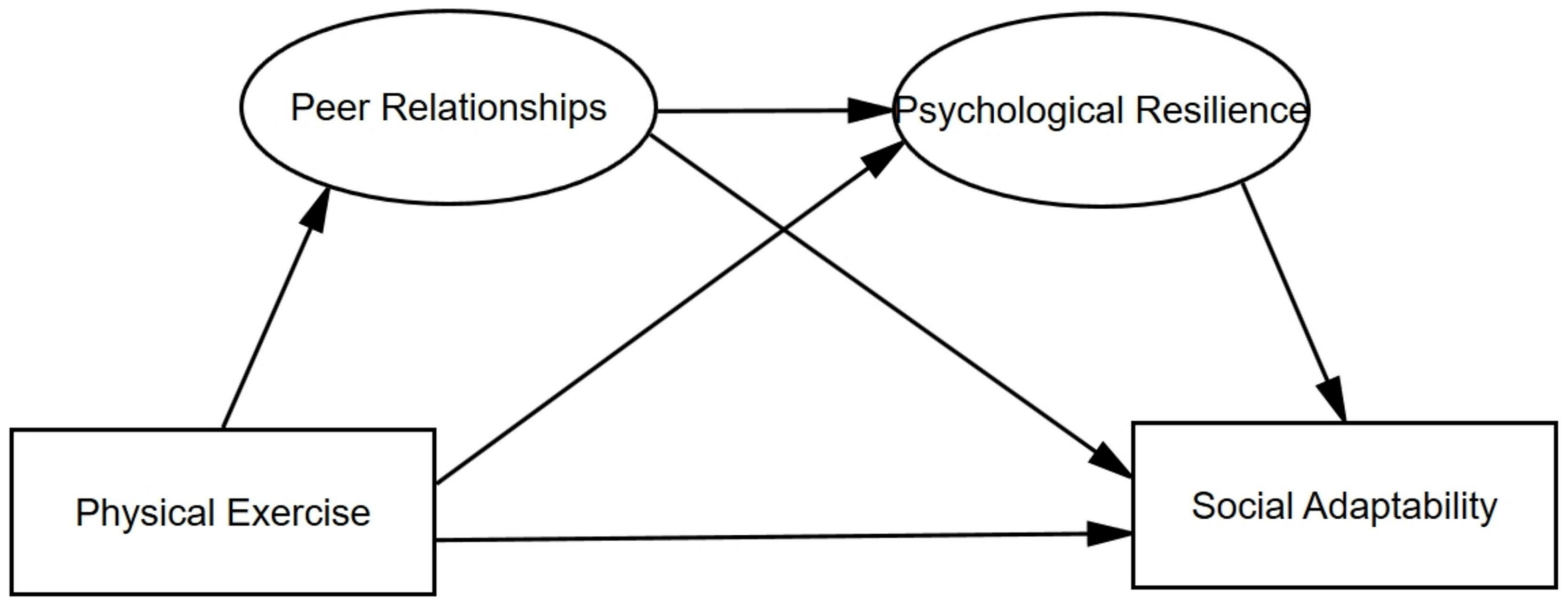
Hypothesis model of physical exercise, peer relationships, psychological resilience, and social adaptability.

In summary, this study, which focuses on college students, proposes the following hypotheses: H1: Physical exercise has a positive effect on college students’ social adaptation; H2: Peer relationships mediate the relationship between physical exercise and social adaptation; H3: Psychological resilience mediates the relationship between physical exercise and social adaptation; H4: Peer relationships and psychological resilience act as chain mediators between physical exercise and social adaptation. Based on this, this study constructed a chain mediation model, as shown in Figure 1.

## Method

### Survey participants

Using convenience sampling, a survey was conducted among college students majoring in various disciplines at select universities in China’s Liaoning, Jilin, and Heilongjiang provinces (which are populous provinces with a diverse student body drawn from across the country, making them representative). To avoid selection bias and ensure the fairness of the study, all participants were students who were not majoring in physical education and had no prior experience in sports training, thereby eliminating any potential interference in the results caused by prior sports training experience. Each participant was required to complete a questionnaire covering demographic information such as gender and academic year. The questionnaire was distributed online; a total of 861 questionnaires were distributed. After excluding invalid responses, 802 valid questionnaires were obtained, representing a response rate of 93.15%, which served as the final dataset for this study. Among them, there were 427 male college students (53.2%) and 375 female college students (46.8%); the number of students in their freshman, sophomore, junior, and senior years were 297 (37.0%), 308 (38.4%), 82 (10.2%), and 115 (14.4%), respectively (Table 1).

**Table 1:** Demographic characteristics of the study participants (*N* = 802).

Demographic variables	Category	Number of people	Percentage (%)
Gender	Male	427	53.2%
	Female	375	46.8%
Grade	Freshman year	297	37.0%
	Sophomore year	308	38.4%
	Junior year	82	10.2%
	Senior year	115	14.4%

### Research tools

#### Physical activity scale

The Physical Activity Rating Scale (PARS-3) revised by Liang (1994) was used to measure the level of physical activity participation. This scale comprises three dimensions: exercise intensity, exercise duration, and exercise frequency. Each dimension is rated on a 5-point scale, and the total score is calculated using the formula “Physical Activity Level = Strength × Time × Frequency,” with a maximum score of 100 points. Based on the total score, the physical activity levels of the study participants were classified into three categories: 19 points or below was classified as low physical activity, 20–42 points as moderate physical activity, and 43 points or above as high physical activity. The Cronbach’s *α* coefficient for this scale was 0.786, confirming that the scale meets reliability standards and ensures data quality.

#### Social adaptation diagnostic scale

The Social Adaptability Diagnostic Scale developed by Zheng (1999) was used. This scale consists of 20 items, such as “I am most afraid of transferring schools or changing classes; whenever I enter a new environment, it always takes me a long time to adapt,” “I find it easy to get along with others whenever I go to a new place,” covering multiple aspects such as adaptation to new environments, interpersonal relationships, attitudes toward learning new knowledge, and performance in special situations. A five-point Likert scale was used, with a maximum score of 100, to measure an individual’s level of social adaptation; a higher score indicates stronger social adaptation ability. In this study, the Cronbach’s alpha coefficient for this scale was 0.904, indicating high reliability and providing reliable and stable data support for the research.

#### Peer relationships scale

The Peer Relationship Scale developed by Zou (1998) was used. It primarily comprises two dimensions: peer acceptance and peer rejection. These dimensions reflect the extent to which an individual is accepted by peers, measure the depth and quality of friendships by assessing feelings of trust and support toward close friends, and evaluate an individual’s self-perception and self-assessment in social contexts, as evidenced by statements such as “I make friends easily at school” and “My classmates all like me in class.” The scale consists of 30 items using a 4-point Likert scale, ranging from “Strongly Disagree” to “Strongly Agree” and scored 1–4 points, respectively. Except for items 1, 3, 4, 7, 11, and 17, all other items are reverse-scored. The total score is out of 120 points; a higher final score indicates better peer relationships. In this study, the Cronbach’s alpha coefficient for this scale was 0.842, indicating high reliability and providing reliable data support for the research.

#### Psychological resilience scale

The Chinese version of the Connor-Davidson Resilience Scale (CD-RISC), revised by Yu Xiaonan and Zhang Jianxin, was used (Yu and Zhang, 2007). The scale consists of 25 items, such as “I do not get discouraged by failure” and “I enjoy challenges.” It is divided into three dimensions: items 2, 3, 4, and 6 constitute the Optimism dimension; items 1, 5, 7, 8, 9, 10, 24, and 25 constitute the Self-Enhancement dimension; and items 11–23 constitute the Resilience dimension. A 5-point Likert scale is used, ranging from “Never” to “Always,” scored from 0 to 4 points respectively, with a maximum total score of 100 points. The sum of the scores for each item constitutes the total score on the Psychological Resilience Scale; a higher score indicates a higher level of psychological resilience. In this study, the Cronbach’s alpha coefficient for this scale was 0.824, indicating high reliability and providing reliable and stable data support for the research.

### Statistical analysis

This study distributed and collected questionnaires via the Wenjuanxing app. A total of 861 questionnaires were distributed, and 861 sets of questionnaire data were collected. The data were entered into Excel 2010, and invalid questionnaires—such as those with missing data or completion times under 180 s—were excluded. Subsequently, SPSS 27.0 was used to perform statistical analysis on the collected data. Outliers were removed based on the criterion that the absolute value of the Z-score for a variable must be greater than 2, ensuring the data met the requirements for a normal distribution. Ultimately, 802 valid questionnaires were obtained, resulting in a response rate of 93.15%. Subsequently, SPSS 27.0 was used to conduct a systematic analysis of the scale and questionnaire data, specifically including reliability and validity tests, descriptive statistics, correlation analysis among variables, and multiple regression analysis. Finally, Amos 29.0 software was used to verify the mediating effects in the research model.

## Results and analysis

### Common method bias

The data for the four variables in this study may be subject to common method bias. Following the approach recommended by Podsakoff et al. (2003), we conducted a validation analysis using Harman’s one-factor test. The results showed that there were 12 factors with eigenvalues greater than 1, and the largest factor explained 26.512% of the variance—well below the critical threshold of 40%. These results indicate that there is no serious common method bias in this study.

### Descriptive statistics and correlation analysis of each variable

An independent samples t-test was used to examine gender differences in college students’ physical exercise, social adaptation, peer relationships, and psychological resilience. As shown in Table 2, significant gender differences were found in physical exercise (*F* = 88.804, *p* < 0.001), social adaptation (*F* = 0.337, *p* < 0.001), peer relationships (*F* = 2.597, *p* < 0.01), and psychological resilience (*F* = 2.651, *p* < 0.001) all showed significant gender differences, with male students scoring higher than female students on all four dimensions.

**Table 2:** Gender differences across variables (*n* = 802).

Dimension	Gender	Number of people	M ± SD	F	*p*
Physical exercise	Male	427	23.14 ± 22.11	88.804	<0.001***
	Female	375	11.08 ± 13.88		
Social adaptability	Male	427	59.94 ± 13.14	0.337	<0.001***
	Female	375	55.68 ± 11.75		
Peer relationships	Male	427	104.89 ± 14.76	2.597	0.004**
	Female	375	101.51 ± 15.90		
Psychological resilience	Male	427	64.71 ± 18.57	2.651	<0.001***
	Female	375	56.93 ± 17.96		

*p < 0.05, **p < 0.01, ***p < 0.001.

A one-way analysis of variance (ANOVA) was conducted to examine grade-level differences in college students’ physical exercise, social adaptation, peer relationships, and psychological resilience. As shown in Table 3, there were no significant differences across grade levels in physical exercise (*F* = 0.285, *p* > 0.05), social adaptation (*F* = 0.695, *p* > 0.05), peer relationships (*F* = 0.156, *p* > 0.05), or psychological resilience (*F* = 1.789, *p* > 0.05).

**Table 3:** Grade-level differences across variables (*n* = 802).

Dimension	Grade	Number of people	M ± SD	F	*p*
Physical exercise	Freshman year	297	15.19 ± 18.21	0.285	0.836
	Sophomore year	308	14.69 ± 18.40		
	Junior year	82	12.60 ± 13.79		
	Senior year	115	15.11 ± 14.34		
Social adaptability	Freshman year	297	56.39 ± 12.35	0.695	0.555
	Sophomore year	308	57.46 ± 12.49		
	Junior year	82	57.79 ± 10.35		
	Senior year	115	58.11 ± 12.95		
Peer relationships	Freshman year	297	102.38 ± 15.57	0.156	0.926
	Sophomore year	308	102.67 ± 15.36		
	Junior year	82	104.05 ± 13.78		
	Senior year	115	102.26 ± 18.35		
Psychological Resilience	Freshman year	297	58.20 ± 18.11	1.798	0.146
	Sophomore year	308	59.69 ± 18.73		
	Junior year	82	61.40 ± 17.82		
	Senior year	115	63.49 ± 19.69		

*p < 0.05, **p < 0.01, ***p < 0.001.

Pearson correlation analysis was used to examine and discuss the relationships among physical exercise, social adaptation, peer relationships, and psychological resilience. As shown in Table 4, physical exercise was found to have significant positive correlations with social adaptation (*r* = 0.551, *p* < 0.01), peer relationships (*r* = 0.425, *p* < 0.01), and psychological resilience (*r* = 0.527, *p* < 0.01). Social adaptation showed significant positive correlations with peer relationships (*r* = 0.623, *p* < 0.01) and psychological resilience (*r* = 0.672, *p* < 0.01). Additionally, a significant positive correlation was observed between peer relationships and psychological resilience (*r* = 0.459, *p* < 0.01). The above results indicate that there are significant positive correlations between all pairs of variables: physical exercise, social adaptation, peer relationships, and psychological resilience. These findings provide preliminary support for testing the hypotheses of subsequent studies and lay the foundation for further exploration of the relationships among these variables.

**Table 4:** Correlation analysis among variables (*n* = 802).

Dimension	M ± SD	1	2	3	4
Physical exercise	14.85 ± 17.78	1			
Social adaptability	57.01 ± 12.35	0.551**	1		
Peer relationships	102.57 ± 15.62	0.425**	0.623**	1	
Psychological resilience	59.37 ± 18.50	0.527**	0.672**	0.459**	1

*p < 0.05, **p < 0.01.

### Multicollinearity issues

Since all variables are significantly correlated, there may be a problem of multicollinearity. Therefore, in this study, covariance diagnostics were performed using standardized analysis. The results indicate that the tolerance values for each predictor variable range from 0.216 to 0.676, all of which are greater than 0.2, while the VIF values range from 1.478 to 4.624, all of which are less than 5. Therefore, there is no issue of multicollinearity in the data (Wen et al., 2018), and the chain mediation analysis can proceed.

### Structural equation modeling

**Figure 2:**
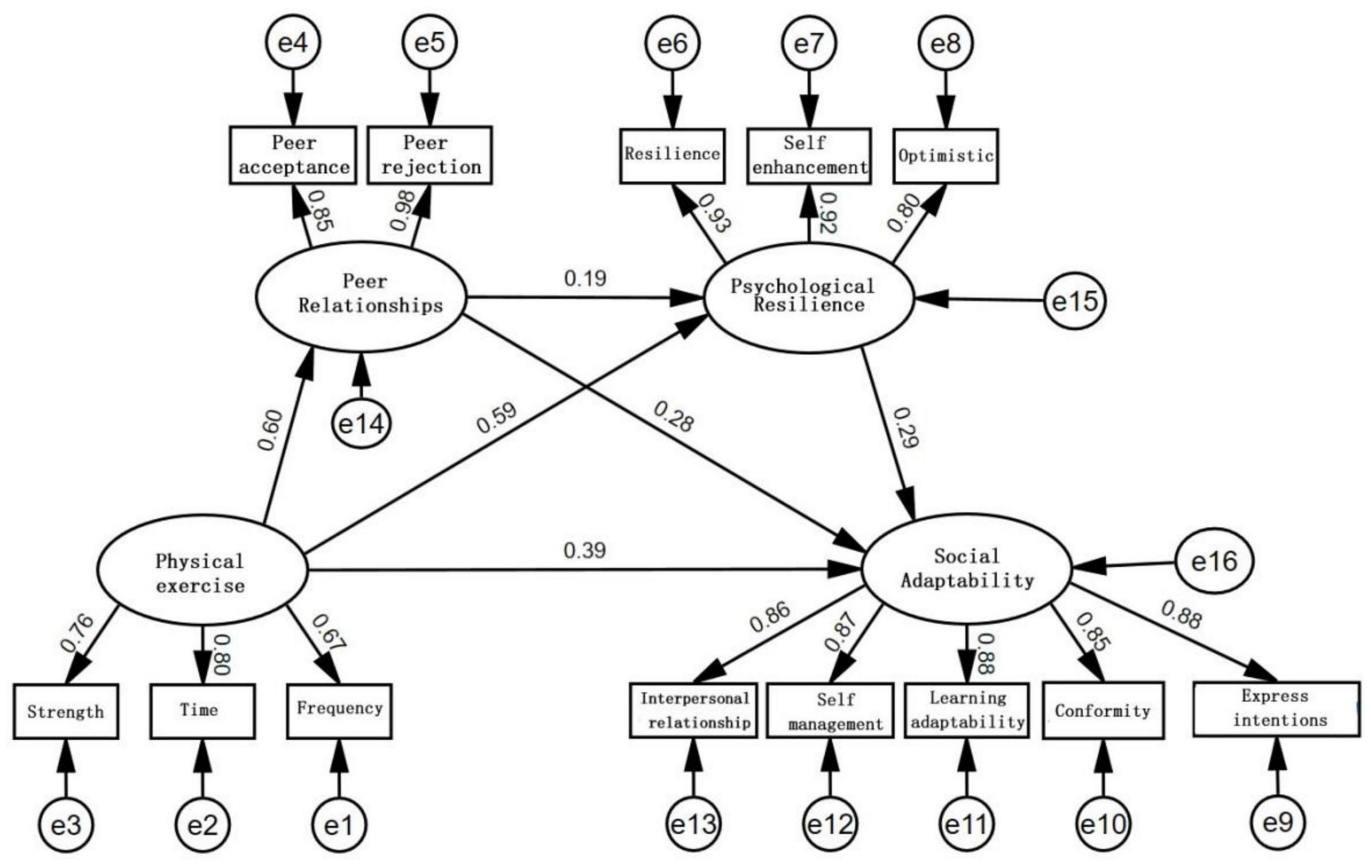
Standardized structural equation model (chain mediation effects of peer relationships and psychological resilience).

According to Wen Zhonglin’s article “Testing Structural Equation Models: Fit Indices and the Chi-Square Criterion,” the chi-square criterion should only be used when N < 1,000 (Wen et al., 2004); therefore, this study reports only the RMSEA results as a measure of absolute fit (Wen and Liang, 2015). This study used Amos 29.0 for modeling and tested a model with physical exercise as the independent variable, social adaptation ability as the dependent variable, peer relationships and psychological resilience as mediating variables, and student gender and grade level as control variables (to keep the model simple, the control variables are not labeled in Figure 2). Using the criteria proposed by Hu and Bentler (1999)—namely, RMSEA < 0.08 and NFI, etc., ≥ 0.90—the Root Mean Square Error of Approximation (RMSEA = 0.054) was less than 0.08, and the remaining model fit indices (NFI, RFI, IFI, CFI, and TLI, with coefficient values of 0.977, 0.970, 0.984, 0.984, and 0.979, respectively) were all greater than 0.9 and within an acceptable range, indicating that the model fits well.

As shown in Figure 2, physical exercise was found to be a positive predictor of peer relationships (*β* = 0.60, *p* < 0.001), psychological resilience (*β* = 0.59, *p* < 0.001), and social adaptation (*β* = 0.39, *p* < 0.001). Peer relationships were found to positively predict psychological resilience (*β* = 0.19, *p* < 0.001) and social adaptation (*β* = 0.28, *p* < 0.001). Additionally, psychological resilience was found to positively predict social adaptation (*β* = 0.29, *p* < 0.001).

### Testing for a mediating effect

To further verify the mediating effects of peer relationships and psychological resilience on the relationship between physical exercise and social adaptation, this study used the Bootstrap method (5,000 repeated random samples) to correct for bias and test for statistical significance. As shown in Table 5, the 95% confidence intervals (CI) for the three path coefficients are as follows: Ind1: Physical Exercise → Peer Relationships → Social Adaptability [0.135, 0.212]; Ind2: Physical Exercise → Psychological Resilience → Social Adaptability [0.122, 0.229]; Ind3: Physical Exercise → Peer Relationships → Psychological Resilience → Social Adaptability [0.018, 0.052]—none of which included 0, and the *p*-values for each path were all less than 0.001. This indicates that peer relationships and psychological resilience each exert a significant independent mediating effect between physical exercise and college students’ social adaptation, and that peer relationships and psychological resilience also exert a significant chained mediating effect between physical exercise and college students’ social adaptation. In summary, physical exercise exerts a significant influence on college students’ social adaptation through peer relationships and psychological resilience (Table 6).

**Table 5:** Model fit indices for the chain mediation model of peer relationships and psychological resilience.

Indicators	RMSEA	NFI	RFI	IFI	CFI	TLI
Fit Index	0.054	0.977	0.970	0.984	0.984	0.979

**Table 6:** Testing the chain mediation effect of peer relationships on psychological resilience.

Pathway	Effect value	SE	*p*	95%CI	
				LB	UB
Ind1: Physical Exercise →Peer Relationships →Social Adaptability	0.172	0.020	<0.001	0.135	0.212
Ind2: Physical Exercise →Psychological Resilience →Social Adaptability	0.171	0.027	<0.001	0.122	0.229
Ind3: Physical Exercise →Peer Relationships →Psychological Resilience →Social Adaptability	0.033	0.009	<0.001	0.018	0.052

## Discussion

### Discussion of gender differences in various variables

Research shows that there are significant gender differences in physical exercise, social adaptation, peer relationships, and psychological resilience. In terms of physical exercise, male college students engage in significantly more exercise than female college students. Most male students have a much greater fondness for sports than female students, particularly in relatively large-scale, highly competitive, and contact sports such as basketball and soccer, where they demonstrate greater athletic talent and self-confidence. This makes it easier for them to achieve a sense of accomplishment, resulting in stronger and more pronounced motivation for physical exercise; Female students, on the other hand, tend to be relatively weaker in these sports, which may require them to put in twice as much effort as their male counterparts to improve. This can severely dampen their enthusiasm and lead to a psychological resistance toward physical exercise (Zhang et al., 2009). In terms of social adaptability, boys generally demonstrate greater social adaptability than girls. This may be related to their upbringing; during their formative years, boys may be given more freedom to explore on their own—such as traveling alone or trying out a wider range of activities—which indirectly enhances their ability to cope with and adapt to unfamiliar environments. Girls, on the other hand, are often reminded to prioritize safety and tend to be better than boys at risk avoidance; however, they are less adept than boys at handling unexpected situations or quickly adapting to new environments (Li and Chen, 2012). In terms of peer relationships, boys tend to have closer bonds with their peers than girls do. Boys’ interactions are more likely to center on shared activities, such as playing video games or playing sports together; They place greater importance on fitting into a specific social group, such as roommates or teammates, and tend to have a relatively large number of acquaintances but relatively few close friends; in contrast, girls may be more inclined to engage in heartfelt conversations with close friends—such as sharing their innermost feelings—and tend to form 1–2 close friendships. Their need to be understood is far greater than that of boys, which leads to significant differences in how they choose and build peer relationships (Deng, 2025a). In terms of psychological resilience, boys generally exhibit higher levels of resilience than girls. This may be due to differing social expectations: boys are expected to be strong, responsible, and less prone to complaining. As a result, when faced with external pressures, most boys tend to “tough it out,” while girls are more often encouraged to express their emotions and seek help. Consequently, the vast majority of boys demonstrate greater psychological resilience than girls (Li, 2020).

### The relationship between physical exercise and social adaptability among college students

Research indicates that there is a significant positive correlation between physical exercise and social adaptation among college students; that is, the higher the level of physical exercise, the stronger the students’ social adaptation. This validates Hypothesis H1 of this study and is consistent with existing research findings (Shan, 2025). Studies have shown that participation in physical activities can strengthen social cohesion and regulate social behavior, thereby significantly improving individuals’ ability to adapt to social roles and their environment (Janssens and Verweel, 2014). The higher the level of participation in physical exercise among college students, the higher their social adaptation ability, and the more likely they are to remain relaxed and at ease in public settings. Conversely, the lower the level of participation in physical exercise, the lower their social adaptation ability, and the more likely they are to experience negative emotions such as embarrassment, anxiety, and tension in public settings (Chen, 2022). That is, the higher the level of participation in physical activities among adolescents, the better their social adaptation levels in terms of interpersonal communication, emotional adaptation, and role integration (Vankim and Nelson, 2013). Consequently, the view that physical exercise influences college students’ social adaptation abilities is recognized by researchers.

### The mediating role of peer relationships

The study found that physical exercise not only directly influences college students’ social adaptation but also affects it indirectly by influencing peer relationships. Peer relationships mediate the relationship between physical exercise and social adaptation, as evidenced by the following: physical exercise significantly and positively predicts peer relationships, and the higher the level of peer relationships, the stronger the students’ social adaptation. This validates Hypothesis H2 of this study and is consistent with the conclusions of previous research (Yu et al., 2023). Research indicates that exercise releases endorphins (pleasure hormones), which can alleviate social anxiety in adolescents, making them more relaxed and confident in social situations and better able to establish positive peer connections (Song, 2024). Long-term, regular physical exercise can promote positive interaction and cooperation among peers, strengthen emotional bonds and mutual support among students, and foster the harmonious and stable development of peer relationships (Dai, 2025). In particular, team sports continuously reinforce students’ communication and interaction, significantly increasing the frequency of peer contact and thereby optimizing peer relationships (van der Pol, 2020). Furthermore, positive and harmonious peer relationships can effectively alleviate feelings of loneliness, promote interpersonal communication and group integration, and consequently significantly enhance social adaptation; conversely, negative peer interactions tend to induce feelings of loneliness and hinder an individual’s social adaptation (Chen et al., 2004). When college students actively participate in physical exercise, they can expand their social interaction contexts, enhance peer communication and collaboration, and establish harmonious and stable peer relationships; in turn, positive peer support and interaction help individuals better adapt to interpersonal interactions, campus life, and the social environment, continuously improving their social adaptation abilities (Dai, 2025). Therefore, peer relationships play a unique mediating role between physical exercise and college students’ social adaptation abilities.

### The mediating role of psychological resilience

The study found that physical exercise not only directly influences college students’ social adaptation abilities but also affects these abilities indirectly by influencing psychological resilience. Psychological resilience mediates the relationship between physical exercise and social adaptation, as evidenced by the following: physical exercise significantly and positively predicts psychological resilience, and higher levels of psychological resilience are associated with stronger social adaptation abilities among college students. This validates Hypothesis H3 of this study and is consistent with the findings of previous research (Tian, 2024). Research indicates that physical exercise plays a positive role in fostering psychological resilience among college students, enhancing their resilience, self-efficacy, and optimism (Li and Liang, 2025). College students who regularly engage in physical exercise demonstrate higher levels of psychological resilience, particularly exhibiting significant advantages in academic performance, adaptation to campus life, and emotional regulation (Shi et al., 2025). Furthermore, students with stronger psychological resilience also exhibit relatively higher levels of social adaptation (Hu, 2019). Students with strong psychological resilience are able to proactively plan their academic and personal lives; they possess strong independence and a sense of autonomy. When facing adversity, they can self-regulate and respond proactively, often maintaining an optimistic and tolerant attitude. Consequently, they perceive less stress and adapt to society more quickly (Yuan, 2019). Active participation in physical exercise helps individuals build interpersonal relationships and gain social support, thereby enhancing their psychological resilience. Strong psychological resilience, in turn, reduces and overcomes feelings of frustration in both academic and daily life, enabling them to engage positively in social interactions, establish closer interpersonal relationships, and further improve their social adaptation abilities (Deng, 2025b). Therefore, psychological resilience plays a unique mediating role between physical exercise and college students’ social adaptation abilities.

### The chain mediation of peer relationships and psychological resilience

This study employed the Bootstrap method (5,000 repeated random samples) to correct for bias. The indirect effect in a chained mediation model is the product of two coefficients; the sampling distribution is necessarily skewed and does not follow a normal distribution. Since the Bootstrap method does not rely on a theoretical normal distribution but instead constructs an empirical sampling distribution through repeated sampling from the original sample, the results are more reliable (Shrout and Bolger, 2002). The results indicate that peer relationships and psychological resilience play a chain-mediating role in the relationship between physical exercise and college students’ social adaptation. Specifically, improved peer relationships lead to enhanced psychological resilience, and peer relationships can significantly and positively predict psychological resilience. This validates Hypothesis H4 of this study and is consistent with the findings of previous research (Zhang, 2024). Research indicates that when students participate in physical exercise, they not only master sport-specific skills but also enhance peer interaction and communication, thereby improving interpersonal skills and social adaptation abilities, which ultimately promotes the formation and development of healthy peer relationships (Jiang, 2024). At the same time, healthy peer relationships enhance individuals’ sense of self-identity, sense of accomplishment, and motivation for voluntary participation (Smith et al., 2006), helping them avoid risks and providing a supportive social environment for the healthy development of psychological resilience (Babaeer et al., 2022). In summary, peer relationships and psychological resilience play a chain-mediating role in the relationship between physical exercise and college students’ social adaptation abilities.

### Limitations and prospects

This study examines the relationship between physical exercise and social adaptation among college students. Theoretically, peer relationships and psychological resilience are treated as mediating variables, with peer relationships representing the social dimension and psychological resilience representing the individual dimension. Physical exercise influences college students’ social adaptation through dual social-individual pathways, thereby providing a theoretical framework for enhancing their social adaptation. In practical terms, the findings of this study offer actionable guidance for improving college students’ social adaptation abilities. By expanding physical education beyond mere skill training to include student interaction and the cultivation of social-psychological competencies, this approach promotes students’ comprehensive development and contributes to the cultivation of high-quality talent for society. However, this study also has some limitations. First, the sample is primarily concentrated in the Chinese provinces of Liaoning, Jilin, and Heilongjiang, resulting in insufficient breadth and representativeness. Future research may consider a broader sampling scope to include more regions; second, as a cross-sectional study, this research can only reflect the covariation among variables and cannot clearly establish causal relationships between physical exercise, peer relationships, psychological resilience, and social adaptation. Consequently, it is difficult to reveal the dynamic trajectory of the long-term effects of physical exercise on college students’ social adaptation. Future research could adopt a longitudinal design to further validate the causal relationships among physical exercise, social adaptation, peer relationships, and psychological resilience.

## Conclusion

Physical exercise, social adaptation, peer relationships, and psychological resilience all showed significant positive correlations with each other in pairs. Physical exercise positively predicts peer relationships, psychological resilience, and social adaptation; peer relationships positively predict psychological resilience and social adaptation; and psychological resilience positively predicts social adaptation. Peer relationships and psychological resilience act as independent mediators between physical exercise and college students’ social adaptation. Peer relationships and psychological resilience act as chain mediators between physical exercise and college students’ social adaptation.

## Data Availability

The raw data supporting the conclusions of this article will be made available by the authors, without undue reservation.
